# Safety of Anticancer Agents Used in Children: A Focus on Their Off-Label Use Through Data From the Spontaneous Reporting System

**DOI:** 10.3389/fphar.2020.00621

**Published:** 2020-05-07

**Authors:** Annamaria Mascolo, Cristina Scavone, Michele Bertini, Simona Brusco, Francesca Punzo, Elvira Pota, Martina Di Martino, Daniela Di Pinto, Francesca Rossi

**Affiliations:** ^1^ Department of Experimental Medicine–Section of Pharmacology “L. Donatelli”, University of Campania “Luigi Vanvitelli”, Naples, Italy; ^2^ Department of Woman, Child and General and Specialist Surgery, University of Campania “Luigi Vanvitelli”, Naples, Italy

**Keywords:** safety, pharmacovigilance, spontaneous reporting system, adverse drug reaction, anticancer agent, pediatric, off-label use

## Abstract

**Background:**

Among factors influencing the higher risk of developing unknown or rare adverse drug reactions (ADRs) among children and adolescents, there is the frequent off-label use of drugs that seems to be very common in pediatric oncological patients. Our study aim to collect and evaluate data on the safety profile of antineoplastic drugs and their off-label use in the pediatrics population using real life data.

**Methods:**

We retrieved Individual Case Safety Reports (ICSRs) with an anticancer agent as suspected drug among those reported through the Campania spontaneous reporting system from 1 January 2013 to 30 September 2019. We classified ICSRs into four off-label categories: “age,” “route of administration,” “weight,” and “therapeutic indication.” We defined an ICSR as an off-label case if it met at least one of the aforementioned categories for at least one of the reported suspected antineoplastic drugs.

**Results:**

A total of 18 ICSRs (7.6%) out of 236 were classified as off-label cases. The median age of patients was 13 years (interquartile range, IQR: 6–16), with 94.4% of cases occurring in male patients. In the classification of the off-label category, 16 ICSRs were categorized according to the “therapeutic indication” and two for the “age.” No case was categorized for the off-label categories “route of administration” and “weight.” The two off-label cases categorized as “age” were both related to the use of brentuximab vedotin for Hodgkin’s lymphoma in patients aged 16 years. Twenty-nine ADRs (1.6 suspected adverse drug reactions per ICSR) were identified among off-label cases. Among ADRs, those reported more than one were diarrhea (N = 3), neutropenia (N = 3), nausea (N = 2), pyrexia (N = 2), and vomit (N = 2).

**Conclusions:**

Our findings showed a low number of ICSRs classified as off-label. The majority of off-label ICSRs were categorized for the “therapeutic indication.” This low number of off-label ICSRs might be largely due to the underreporting phenomenon, which is a major limit in pharmacovigilance. Therefore, we believe that spreading pharmacovigilance knowledge and awareness might improve this aspect.

## Introduction

Children and adolescents present an higher risk of developing unknown or rare adverse drug reactions (ADRs) compared to the adult population (Pellegrino et al., 2013). This is due to a different and immature pharmacokinetic and pharmacodynamics profile (i.e. different volumes of distribution and activities of drug‐metabolizing enzymes/transporters), uncertainties on the long-term drug risk-benefit profile, and frequent off-label use of drugs (Lerose et al., 2012; Shebley et al., 2019; Sultana et al., 2019). Indeed, medicines authorized for the use in pediatric age are still very few, due to major barriers existing in testing and licensing medications for children such as small market size and fewer chronic illnesses and the resulting difficulty in enrolling a sufficient number of pediatric patients (Milne and Bruss, 2008). Consequently, many drugs are used in the pediatric population on an off-label basis, erroneously assuming and translating the efficacy and safety of a compound, from adults to pediatric age (Napoleone, 2010). A pediatric study from a national ADR database examined the contribution of off-label prescribing by age to the occurrence of ADRs. This study showed that off-label prescribing is associated with a large number of serious ADRs, including fatal cases (Aagaard and Hansen, 2011). In the literature, among drugs most frequently prescribed off-label there are anticonvulsants, antibiotics, rhinologics, antitussives, and gastrointestinal medicines (Gill et al., 1995; Turner et al., 1999; Clarkson et al., 2001; Horen et al., 2002; Schirm et al., 2004; Ufer et al., 2004). In Italy, a study found that paracetamol and beclomethasone were the substances most often used off-label in children (Pandolfini et al., 2002). However, the off-label use of oncological drugs also seems to be very common in the pediatric age (Conroy et al., 2003). This specific clinical area was recently revolutionized by the development of targeted new agents that represent a more effective and safe treatment for children with malignancies. Nevertheless, even though the safety profile of any kind of anticancer treatments (chemotherapeutic and targeted agents) in children is similar to that observed in adults, the severity, frequency, nature, and presentation of adverse drug reactions (ADRs) can differ (Adamson, 2015). Studies have assessed the quota of the off-label use of anticancer drugs in children (Conroy et al., 2003; Cuzzolin et al., 2006; Shah et al., 2007), but data on their safety are scarce. Therefore, considering the growing use of off-label drugs in pediatric clinical practice, including the oncology setting, and safety concerns related to this kind of use as well as the different safety profile of medicines in children and adolescents compared to that observed in adult patients, we have examined the individual case safety reports (ICSRs) of suspected ADRs concerning antineoplastic drugs used in pediatric patients (aged less than 18 years) sent to the Italian pharmacovigilance Spontaneous Reporting System in Campania Region. The aim of this study was to collect and evaluate data on the safety profile of antineoplastic drugs used in pediatrics in a real life setting and to evaluate the amount of off-label use among ICSRs reporting cancer drugs-induced ADRs.

## Methods

### Data Source

Individual Case Safety Reports (ICSRs) were retrieved among those recorded in the spontaneous reporting system from 1 January 2013 to 30 September 2019. The Italian pharmacovigilance spontaneous reporting database (Rete Nazionale di Farmacovigilanza–RNF) has been established in 2001 by the Italian Medicines Agency (AIFA). The RNF collects all ICSRs spontaneously reported or deriving from active pharmacovigilance projects or observational studies. ICSRs are sent to the RNF by a Responsible Person for pharmacovigilance, nominated in each national healthcare institution. As Pharmacovigilance Regional Center we have full access only to ICSRs sent to the RNF by Campania Region, South of Italy. Therefore, only regional ICSRs were evaluated.

### ICSRs Selection

ICSRs with at least one antineoplastic drug were identified from the 2nd level of the Anatomical Therapeutic Chemical (ATC) Classification System (L01—antineoplastic agents). We selected only cases emerged in patients aged less than 18 years old. ICSRs not reporting the patient’s age were excluded from the analysis. Moreover, we excluded ICSRs in which the antineoplastic drug was not used for oncologic therapeutic indication or in which there were inconsistencies. All ICSRs were checked for duplicate by crossing information such as gender, age, date of birth, suspected drugs, date of onset, and type of ADRs among ICSRs. Whether it was found that two or more reports were related to the same patient and the ADRs were developed in the same time frame, we combined the information in just one ICSR.

### Descriptive Analysis of ICSRs

ICSRs were described according to gender, median age, seriousness and outcome of the ADR, type of reporter, causality assessment, and number of reported suspected drugs. These data were provided for all ICSRs included in the study, whether they were reporting an off-label use or not. We also described the number and percentage of missing data (e.g. gender, outcome, reporter). To identify the off-label use, ICSRs were individually screened, case-by-case, by a team composed of clinicians and pharmacists experienced in pharmacovigilance and clinical research. Off-label ICSRs were classified into four categories: “age” defined as the administration of a prescription drug outside the age range for which the product was licensed; “route of administration” defined as the use of an alternative route of administration other than that licensed; “weight” administration of drugs for weights for which the product was not licensed; “indication” defined as the prescription of a drug for therapeutic indications that were not licensed. An ICSR was defined as an off-label case if it met at least one of the aforementioned categories for at least one of the reported suspected antineoplastic drugs.

To identify off-label ICSRs, we used as a reference of licensing information, the Summary of Product Characteristics (SmPC) published by the AIFA, and the pediatrics list of antineoplastic products allowed to be used off-label by the Italian law 648/96. This Law, which was approved on December 23^rd^ 1996, regulated the off-label prescription of medicines at the charge of the National Health System (NHS). The inclusion of medicines in the official list of the Italian law 648/96 is subject to the approval by the AIFA Technical Scientific Advisory Commission (CTS) and it is reserved for innovative drugs authorized abroad, but not in Italy, drugs which have not yet received an authorization but have undergone clinical trials, and drugs to be used for a therapeutic indication different from the authorized one. In this list are also contained drugs that can be used for rare oncological diseases (Italian Medicine Agency, 2018). Moreover, to evaluate the preventable quota of ADRs we screened off-label ICSRs to identify risk factors that could have contributed to the onset of ADRs (e.g. drug-drug interactions, drug-disease interaction, drug-herbs interactions, non-compliance, documented hypersensitivity). To check for drug interactions we used a large independent medicine information website (Drugs.com, 2020) (Drugs.com). Finally, to evaluate the performance of our pharmacovigilance system in the off-label setting, which requires the codification of the term “off-label use” with the codification of the ADR, we have also checked how many ICSRs identified as off-label had the “off-label use” codified in the RNF. The characteristics of the off-label cases were summed up in a Table.

The seriousness of ADRs was codified following the International Council on Harmonization E2D guidelines (ICH E2D Post-approval safety data management | European Medicines Agency, 2018), whereas the outcome was categorized into six categories (recovered, improvement, resolution with sequelae, unchanged clinical condition, death, and not available) based on the national law. To establish the strength of a correlation between the suspected drug and the ADR, the Naranjo algorithm was used (Naranjo et al., 1981). All scores that ranged between possible and certain were considered for causality.

Non-off-label ICSRs were classified for the suspected antineoplastic protocol and for the general therapeutic indication which was divided into three categories (hematological tumor, solid tumor, and bone marrow transplant). Moreover, a specification of therapeutic indications among non-off-label ICSRs was performed.

Adverse events were tabled according to the four most reported preferred terms (PTs) and the corresponding System Organ Class (SOC) for non-off-label ICSRs. The time to event defined as the difference in days between the onset date of the adverse event and the date of the administration of the last drug of a specific antineoplastic protocol was independently computed for ICSRs classified as off-label and those classified as non-off-label. A boxplot of time to events was generated.

### Compliance With Ethical Standards

Safety data deriving from the Italian spontaneous reporting system are anonymous and in compliance with the ethical standard. Therefore, no further ethical measure was required.

## Results

### General Characteristics of ICSRs

Between January 2013 to September 2019, 7,610 ICSRs with an antineoplastic drug as suspected drug were sent to the RNF from the Campania Region, of which 253 (3.3%) occurred in pediatric patients. Campania Region contributed for the 44.3% of national ICSRs on anticancer agents used in children (N = 571). Among regional pediatric ICSRs, we screened for the evaluation of the off-label use a total of 236 ICSRs (92.9%), of which 47 ICSRs (19.9%) were from non-interventional studies. Twelve ICSRs were excluded due to the use of the suspected antineoplastic drug in a non-oncology setting and five due to inconsistencies. The non-oncological therapeutic indications were bone marrow transplant due to aplastic anemia, osteopetrosis, or not specified for fludarabine, methotrexate, cyclophosphamide, busulfan, and anti-thymocyte globulins (N = 9), nephrotic syndrome for rituximab (N = 2), and ulcerative colitis for methotrexate (N = 1). A total of 218 (92.4%) ICSRs out of 236 were classified as non-off-label cases. The median age of patients was 9 years (interquartile range, IQR: 6–12), with 64.7% of cases occurring in male patients. Most ICSRs were reported as not serious (112; 51.4%). Among ICSRs classified as serious, 25.2% was related to clinically significant conditions and 23.4% to hospitalizations. Most ICSRs reported a positive outcome classified as recovered (60.5%) or improvement (29.8%). The main reporter was the physician with 211 (96.8%) out of 218 ICSRs. The causality assessment was possible for 211 (96.8%) and probable for 6 (2.7%) ICSRs. Characteristics of cases were presented in **Table 1**. Most ICSRs (85.3%) reported more than one antineoplastic drug as suspected drug (**Table 1**). The main general therapeutic indication for the administration of antineoplastic protocols suspected to be responsible for the ADRs were hematological tumors (160; 78.4%), followed by bone marrow transplant (53; 26.1%), and solid tumors (5; 3.7%) (**Table 2**). A specification of the suspected antineoplastic protocol for the main general therapeutic indication is shown in **Table 2**. Among hematological tumors, the most reported was acute lymphocytic leukemia (105; 65.6%), followed by Burkitt lymphoma (19; 11.9%) and acute myeloid leukemia (9; 5.6%) (**Table 3**). Among solid tumors, there was a case of glioblastoma multiforme, osteosarcoma, embryonal rhabdomyosarcoma, metastatic kidney cancer, and breast cancer.

**Table 1:** Demographic characteristics and distribution for the type of reporter, seriousness and outcome of adverse drug reactions, causality assessment, and number of the reported suspected drugs of all pediatric ICSRs and those reporting or not reporting an off-label use that involve antineoplastic drugs as suspected drugs among those recognized in the Campania spontaneous reporting system from January 2013 to September 2019.

Variable	Level	ICSRs reporting an off-label use (N = 18)	ICSRs not reporting the off-label use (N = 218)	All ICSRs (N = 236)
Gender	Female	1 (5.6)	73 (33.5)	74 (31.3)
	Male	17 (94.4)	141 (64.7)	158 (66.9)
	Not available	–	4 (1.8)	4 (1.8)
Age	Median (IQR)	13 (6–16)	9 (6–12)	9 (6–13)
Seriousness	Serious–other clinically significant condition	3 (16.7)	55 (25.2)	58 (24.6)
	Serious–hospitalization	2 (11.1)	51 (23.4)	53 (22.4)
	Serious–life threatening	2 (11.1)	–	2 (0.9)
	Not serious	11 (61.1)	112 (51.4)	123 (52.1)
Outcome	Improvement	11 (61.1)	65 (29.8)	76 (32.2)
	Unchanged clinical condition	–	9 (4.1)	9 (3.8)
	Not available	–	10 (4.7)	10 (4.2)
	Recovered	7 (38.9)	132 (60.5)	139 (58.9)
	Resolution with sequelae	–	2 (0.9)	2 (0.9)
Time to event	Median (IQR)	11 (3.25–14.5)	9 (4–15)	9 (4–15)
Reporter	Physicians	17 (94.4)	211 (96.8)	228 (96.6)
	Pharmacist	–	1 (0.5)	1 (0.4)
	Other health care professional	–	5 (2.3)	5 (2.1)
	Patient/Citizen or other non-healthcare professional figure	1 (5.6)	1 (0.5)	2 (0.9)
Causality	Possible	18 (100.0)	211 (96.8)	229 (97.0)
	Probable	–	6 (2.7)	6 (2.5)
	Doubt	–	1 (0.5)	1 (0.5)
Number of reported suspected drugs	>1	13 (72.2)	186 (85.3)	199 (84.3)
	1	5 (27.8)	32 (14.7)	37 (15.7)

**Table 2:** Number of non-off-label ICSRs distributed for the suspected antineoplastic protocols and the general therapeutic indication in the setting of the Campania spontaneous reporting system—January 2013 to September 2019.

Cancer and suspected antineoplastic protocols	Number of ICSRs	Percentage (%)
***Hematological tumor***	***160***	***78.4***
Daunorubicin	2	1.3
Daunorubicin + vincristine	13	8.1
Daunorubicin + vincristine + pegaspargase	5	3.1
Daunorubicin + vincristine + methotrexate	6	3.8
Daunorubicin + vincristine + crisantaspase	1	0.6
Daunorubicin + vincristine + methotrexate + pegaspargase	15	9.4
Daunorubicin + cyclophosphamide + vincristine	1	0.6
Daunorubicin + vindesine + pegaspargase + methotrexate + ifosfamide	1	0.6
Doxorubicin + vincristine	2	1.3
Doxorubicin + vincristine + pegaspargase	1	0.6
Doxorubicin + vincristine + L-asparaginase	1	0.6
Arsenic trioxide	4	2.5
Cyclophosphamide	3	1.9
Cyclophosphamide + methotrexate	1	0.6
Cyclophosphamide + methotrexate + citarabine	1	0.6
Cyclophosphamide + methotrexate + vincristine	3	1.9
Cyclophosphamide + citarabine	3	1.9
Cyclophosphamide + citarabine + vincristine	2	1.3
Cyclophosphamide + vincristine	4	2.5
Cyclophosphamide + vincristine + rituximab + methotrexate	3	1.9
Cyclophosphamide + doxorubicin + methotrexate + vincristine	1	0.6
Cyclophosphamide + etoposide + citarabine	1	0.6
Cyclophosphamide + 6-mercaptopurine + citarabine	3	1.9
Citarabine	6	3.8
Citarabine + idarubicin	2	1.3
Citarabine + clofarabine	1	0.6
Citarabine + 6-mercaptopurine	5	3.1
Citarabine + 6-mercaptopurine + methotrexate	2	1.3
Citarabine + pegaspargase	1	0.6
Etoposide + cyclophosphamide + clofarabine	1	0.6
Etoposide + citarabine	1	0.6
Etoposide + citarabine + ifosfamide + vincristine	2	1.3
Etoposide + citarabine + vindesine	1	0.6
Idarubicin + etoposide + citarabine	5	3.1
Idarubicin + pegaspargase + vincristine	2	1.3
Idarubicin + vincristine	1	0.6
Imatinib	1	0.6
Imatinib + doxorubicin + vincristine + L-asparaginase	11	6.9
Imatinib + methotrexate + vindesine	2	1.3
6-mercaptopurine	8	5.0
6-mercaptopurine + methotrexate	8	5.0
Mitoxantrone	1	0.6
Pegaspargase	3	1.9
Pegaspargase + cyclophosphamide + citarabine + vincristine + metotrexate	1	0.6
Pegaspargase + cyclophosphamide + vincristine + methotrexate	3	1.9
Pegaspargase + citarabine + vincristine + 6-mercaptopurine + methotrexate	2	1.3
Pegaspargase + vindesine + ifosfamide + methotrexate	1	0.6
Pegaspargase + vindesine + methotrexate	1	0.6
Rituximab + cyclophosphamide + doxorubicin	1	0.6
Rituximab + daunorubicin + methotrexate + citarabine	1	0.6
Rituximab + daunorubicin + cyclophosphamide + vincristine + methotrexate + citarabine	1	0.6
Rituximab + ifosfamide + vincristine + methotrexate + citarabina + etoposide	2	1.3
Rituximab + ifosfamide + vincristine + methotrexate + citarabine + cyclophosphamide	4	2.5
Rituximab + vincristine + methotrexate + citarabine + cyclophosphamide	2	1.3
***Solid tumor***	***5***	***3.7***
Cyclophosphamide + epirubicin + 5-fluorouracil	1	20.0
Dactinomycin + vincristine + ifosfamide	1	20.0
Dactinomycin + vincristine + doxorubicin	1	20.0
Methotrexate	1	20.0
Temozolomide	1	20.0
***Bone marrow transplant***	***53***	***26.1***
Busulfan	2	3.8
Busulfan + melphalan + cyclophosphamide + methotrexate	4	7.5
Busulfan + melphalan + cyclophosphamide	2	3.8
Busulfan + melphalan	27	50.9
Busulfan + fludarabine + thiotepa	7	13.2
Cyclophosphamide + thiotepa	1	1.9
Cyclophosphamide + thiotepa + methotrexate	5	9.4
Melphalan + fotemustine + citarabine + etoposide	5	9.4

**Table 3:** Number of non-off-label ICSRs with an antineoplastic protocols as suspected drugs distributed for the specific therapeutic indication in the setting of the Campania spontaneous reporting system—January 2013 to September 2019.

Therapeutic indications	Total	%
**Hematological tumors**	**160**	**73.4**
Acute lymphocytic leukemia	105	65.6
Burkitt lymphoma	19	11.9
Acute myeloid leukemia	9	5.6
Relapsed leukemia	9	5.6
Relapsed acute lymphocytic leukemia	8	5.0
T-cell acute lymphoblastic leukemia	6	3.7
Relapsed acute myeloid leukemia	3	1.9
Non-Hodgkin’s lymphoma	1	0.6
**Solid tumors**	**5**	**2.3**
Glioblastoma multiforme	1	20.0
Osteosarcoma	1	20.0
Embryonal rhabdomyosarcoma	1	20.0
Metastatic kidney cancer	1	20.0
Breast cancer	1	20.0
**Bone marrow transplant**	**53**	**24.3**

### Safety of Antineoplastic Drugs in Non-Off-Label ICSRs

We observed a total of 420 ADRs (1.9 suspected adverse drug reactions per ICSR) since more than one ADR could be reported in each ICSR. A total of 130 (31.0%) out of 420 ADRs belongs to the SOC “Gastrointestinal disorders,” followed by the SOC “Blood and lymphatic system disorders” (64; 15.2%), the SOC “General disorders and administration site conditions” (52; 12.4%), the SOC “Respiratory, thoracic, and mediastinal disorders” (28; 6.7%), and the SOC “Hepatobiliary disorders” (26; 6.2%). Accordingly, the most reported adverse events were: vomit (29/130; 22.3%), neutropenia (19/64; 29.7%), pyrexia (18/52; 34.6%), elevated transaminases (19/26; 73.1), thrombocytopenia (16/64; 25.0%), diarrhea (14/130; 10.8%), abdominal pain (14/130; 10.8%), nausea (13/130; 10.0%), anemia (13/64; 20.3%), mucositis (12/52; 23.1), and oropharyngeal pain (8/28; 28.6%). The four most reported adverse events classified per SOC are listed in **Supplementary Table 1**. The median time to event was 9 days (interquartile range [IQR]: 4–15) for the ICSRs classified as non-off-label.

### Off-Label ICSRs

A total of 18 ICSRs (7.6%) out of 236 were classified as off-label cases, none of these ICSRs was from non-interventional studies. The median age of patients was 13 years (interquartile range, IQR: 6–16), with 94.4% of cases occurring in male patients. Most ICSRs were reported as not serious (11; 61.1%). Among ICSRs classified as serious, the most reported criterion for seriousness was “serious–other clinically significant condition” (3; 16.7%), followed by “serious–hospitalizations” (2; 11.1%) and “serious–life-threatening” (2; 11.1%). All ICSRs reported a positive outcome classified as recovered (7; 38.9%) or improvement (11; 61.1%). The main reporter was the physician with 17 (94.4%) out of 18 ICSRs. Causality assessment was possible for all ICSRs. The characteristics of off-label cases were presented in **Table 1**.

In the classification of the off-label category, 16 ICSRs were categorized according to the “therapeutic indication” and two for the “age.” No case was categorized into the off-label categories “route of administration” and “weight” (**Table 4**). For off-label cases due to the indication, four (2.2%) reported the use of fotemustine for bone marrow transplant, three (1.7%) the use of ifosfamide for acute lymphocytic leukemia, three (1.7%) the use of bortezomib for acute lymphocytic leukemia, two (1.1%) the use of cyclophosphamide for the acute myeloid leukemia, two (1.1%) the use of melfalan for the Wilms’ tumor, one (0.6%) the use of docetaxel for relapsed osteosarcoma, and one (0.6%) the use of gemcitabine and vinorelbine for Hodgkin’s lymphoma. The two off-label cases categorized as “age” were both related to the use of brentuximab vedotin for Hodgkin’s lymphoma in patients aged 16 years. These ICSRs on brentuximab vedotin were not considered duplicates as they are related to different ADRs reported in different time frames. A total of 29 ADRs (1.6 suspected adverse drug reactions per ICSR) were identified among cases. Among ADRs, those reported more than one were diarrhea (N = 3), neutropenia (N = 3), nausea (N = 2), pyrexia (N = 2), and vomit (N = 2) (**Table 4**). The median time to event was 11 days (IQR: 3.25–14.5) for the ICSRs classified as off-label.

**Table 4:** Characteristics of off-label ICSRs reporting an antineoplastic drug as suspected drug among those reported in the Campania spontaneous reporting system from January 2013 to September 2019.

Number of ICSRs	Antineoplastic drug used off-label	Approved indication*	Off-label use in the ICSR	Other suspected antineoplastic drugs	Category	Number of ADRs	Type of ADR *(Preferred term)*
2	Cyclophosphamide	Cytostatic treatment, acute lymphocytic leukemia, non-Hodgkin’s lymphoma, Hodgkin’s lymphoma, neuroblastoma, rhabdomyosarcoma, low-grade glioma, Ewing’s sarcoma, medulloblastoma osteosarcoma, hepatoblastoma	Acute myeloid leukemia	6-mercaptopurine and citarabine Methotrexate	Indication	2	Nausea, stomatitis
2	Melfalan	Sarcoma, multiple myeloma, malignant melanoma, advanced ovarian cancer, neuroblastoma, Ewing’s sarcoma	Wilms’ tumor	Carboplatin and etoposide	Indication	4	Diarrhea, abdominal pain, mucositis, pyrexia
4	Fotemustine	Malignant melanoma, primitive cerebral tumour	Bone marrow transplant	Citarabine and etoposide	Indication	8	Constipation, vomit, pyrexia, septic shock, diarrhea, increased creatinine, fluid retention, pneumonia
3	Ifosfamide	Bronchial cancer, ovarian cancer, breast cancer, testicular tumor, sarcoma, pancreatic cancer, endometrial cancer, malignant lymphoma, hypernephroma, Wilms’ tumor, hepatoblastoma, neuroblastoma, rhabdomyosarcoma, Ewing’s sarcoma, osteosarcoma	Acute lymphocytic leukemia	Daunorubicin and vindesine (2)	Indication	4	Neutropenia (2), rash, productive cough,
1	Docetaxel	Breast cancer, prostatic cancer, gastric adenocarcinoma, non-small cell lung cancer, head and neck cancer	Relapsed osteosarcoma	None	Indication	3	Generalized edema, antithrombin III decreased, hypoalbuminemia
3	Bortezomib	Multiple myeloma, mantle cell lymphoma	Acute lymphocytic leukemia	Vincristine (1) Vincristine and pegaspargase (1)	Indication	4	Neurotoxicity, anemia, hypercholesterolemia, hypertriglyceridemia
2	Brentuximab vedotin	Hodgkin’s lymphoma, systemic anaplastic large cell lymphoma, cutaneous T cell lymphoma	Hodgkin’s lymphoma	None	Age	3	Nausea, vomit, diarrhea
1	Gemcitabine	Bladder cancer, pancreatic adenocarcinoma, ovarian cancer, non-small cell lung cancer, breast cancer	Hodgkin’s lymphoma	Ifosfamide	Indication	1	Neutropenia
	Vinorelbine	Non-small cell lung cancer, metastatic breast cancer, aggressive fibromatosis, rhabdomyosarcoma					

*Based on the Summary of Product Characteristics (SmPC) and the pediatric list of medicinal products (Italian Law 648/96).

In 11 (61.1%) out of 18 ICSRs there were drug-drug interactions. In four ICSRs more than one interaction was reported. In the majority of cases (N = 9), we identified an interaction among chemotherapy drugs and the development of events like nausea, abdominal pain, vomit, pyrexia, constipation, cough, pneumonia, neutropenia, fever, septic shock, and mucositis. In one ICSR, the interaction was between bortezomib and vincristine and the onset of neurotoxicity. In five ICSRs the interaction was between an anticancer agent and another drug. Specifically, four ICSRs reported an interaction between etoposide and fluconazole with the development of gastrointestinal events (vomit, constipation, mucositis, diarrhea, and abdominal pain), pyrexia, and septic shock, and one ICSR reported an interaction between sulfamethoxazole/trimethoprim and methotrexate with the development of stomatitis (data not shown).

One out of 18 ICSRs had the term “off-label use” codified among ADRs in the RNF. This ICSR reported the onset of neurotoxicity after the administration of bortezomib for acute lymphocytic leukemia in a 6-year-old female patient (the other reported suspected antineoplastic drug was vincristine).

## Discussion

This is the first study in our Region that aims to describe the safety profile of antineoplastic drugs and their off-label use in the pediatric population by extracting data from the Campania region (Italy) spontaneous reporting system. In Italy, the cancer incidence rates in children and adolescents are slightly higher in comparison to other developed Countries, but relatively consistent among different Italian areas. Moreover, the efficacy of antineoplastic protocols has improved constantly since the Seventies, and recent findings have confirmed this trend in all age groups, and also for rare types of cancer with a very poor prognosis (Pisani et al., 2013). In our study, we provided for the first time “real-world evidence” on off-label ICSRs in the setting of pediatric oncology, which is characterized by the absence of standard pediatric prescribing information and safety-efficacy data on drug use. Moreover, we were able to provide pharmacovigilance data that are nowadays more needed on this topic, considering that the off-label drug use is a patient safety-issue highly associated with an increased risk of ADRs, especially in children treated with anticancer drugs (Gore et al., 2017). Approximately 3% of our ICSRs reporting at least one antineoplastic drug as a suspected drug was related to pediatric patients. This low percentage is in accordance with data derived from Italian prescription registries that show a low consumption of drugs belonging to the ATC “L–Antineoplastic and immunomodulating agents” (0.6%) among children (OsMed, 2018). Moreover, cancer is a rare event in children, representing only 2% of all cancer cases in the world (Davidoff, 2010).

In our study, the 2.9% of the ICSRs was excluded due to inconsistencies, which are a limiting factor for case evaluation and for the detection of safety signals in a spontaneous reporting system. In fact, a good report quality is fundamental for the risk management of marketed drugs and is the basis of an effective drug safety monitoring and data utilization in pharmacovigilance (Niu et al., 2019). Most of our pediatric cases were classified as non-off-label with a mean age of 9 years (IQR: 6–12) in accordance with data that showed a higher distribution of pediatric oncology patients ranged between 2 and 12 years (van den Berg and Tak, 2011). In spite of the higher use of antineoplastic agents (as ATC L) in female children (about 60%) (OsMed, 2018), we found that more than 50% of ICSRs were related to male patients. Even though generally female patients (both children and adults) are more likely to experience ADRs than males (Scavone et al., 2017; Scavone et al., 2018), as also confirmed by a study that analyzed data collected within VigiBase, the WHO global database of ICSRs (Watson et al., 2019), other pharmacovigilance studies, involving both adult and pediatric population and different therapeutic class of drugs, reported a higher incidence of ADRs in males compared to females (Montastruc et al., 2002; Ferrajolo et al., 2014; Cliff-Eribo et al., 2016; Lombardi et al., 2019). Furthermore, a recent observational study reported no difference in ADRs between male and female (Singh et al., 2017).

Forty-nine percent of our cases were classified as serious. Accordingly, it has been estimated that 40% of cancer patients suffer life-threatening or permanently disabling severe ADRs, which tend to be even more frequent and severe in children (Ross et al., 2011). However, in most of our ICSRs (90%), there was a positive clinical outcome such as recovered or improvement in accordance with another Italian retrospective analysis based on reports of suspected ADRs, which found that most ADRs occurred in pediatric patients have a positive outcome (Lombardi et al., 2018).

The main reporter was the physician in accordance with the highly specialized environment in which these drugs are used and the particular attention that the medical team has for this subpopulation. Moreover, based on our experience, the physicians is the reporter most sensitive to pharmacovigilance in our region (Sessa et al., 2017; Sessa et al., 2018b; Mascolo et al., 2019).

As reported in the literature, we found that the most reported were hematological tumors (Davidoff, 2010). Specifically, the most reported was the acute lymphocytic leukemia (105; 65.6%), which is described as the most common cancer diagnosed in children (Cooper and Brown, 2015). Accordingly, a registry-based study conducted by the Association Italian Pediatric Hematology-Oncology (AIEOP) on data of Campania Region showed that cancers diagnosed in children were prevalently onco-hematological tumors and among them the acute lymphocytic leukemia was the most reported. Moreover, among solid tumors the most reported were those related to the central nervous system (Indolfi et al., 2016). In the evaluation of antineoplastic protocols, in accordance with a review of medicines for the treatment of common tumors in children published by the World Health Organization (WHO) and the ESMO clinical practice guidelines on hematological malignancies, we observed that anticancer agents like cyclophosphamide, daunorubicin, doxorubicin, pegaspargase, vincristine, etoposide, and imatinib were reported for the treatment of hematological tumors, while dactinomycin, ifosfamide, vincristine, cyclophosphamide, temozolamide, and methotrexate for the treatment of solid tumors (ESMO, 2020; WHO, 2011). A published report on the drug use showed that the most prescribed anticancer agents, in Campania region, belong to the class of monoclonal antibodies, followed by tyrosine kinase inhibitors, and cytostatic antineoplastic agents (OsMed, 2017).

In our study, we found a low number of ICSRs classified as off-label (7.6%). This may be related to the underreporting phenomena, which is a major limitation in pharmacovigilance (Biagi et al., 2013), mainly associated with the fear of repercussions for the reporting of medical errors especially if we are in the field of the off-label use (Castel et al., 2015). Global reporting levels are estimated to be no more than 5–10% of the real incidence in spite of efforts and initiatives to institute a regulatory framework for ADR reporting (Hazell and Shakir, 2006; González-Rubio et al., 2011). In fact, underreporting still is a huge problem in pharmacovigilance that impairs the generation of fast and dynamic safety alerts and the possibility to drawn real conclusions about drug safety, resulting in a reduced quality of patient’s care (González-Rubio et al., 2011). Based on our results, age and gender in off-label cases were consistent with those observed with non-off-label cases. Some studies reported a higher occurrence of ADRs in children exposed to cancer chemotherapy (Posthumus et al., 2012; Bellis et al., 2014). Mitchell et al. estimated 22% of ADRs occurring in pediatric oncology patients compared to the 2% in non-oncology patients (Mitchell et al., 1988). Moreover, a study based on prescription data showed that the risk of developing ADRs is higher when drugs are used off-label compared to their authorized use. This result was largely driven by the occurrence of ADRs in the oncological practice, where the risk of ADRs was found higher than in non-oncological areas (Bellis et al., 2014).

In our off-label cases, most ICSRs were categorized off-label due to the therapeutic indication. Specifically, four ICSRs reported the use of fotemustine for bone marrow transplants. The combination of fotemustine plus etoposide, cytarabine, and melphalan has been evaluated in the literature as a feasible and safe therapeutic alternative in adult patients with relapsed/refractory lymphoma undergoing autologous stem-cell transplantation (auto-SCT) (Musso et al., 2010). Three ICSRs reported the use of ifosfamide for acute lymphocytic leukemia. In the literature, data on the efficacy and safety of ifosfamide for acute lymphocytic leukemia in children dated back to ‘90s (Reiter et al., 1992; Crooks and Sato, 1995). Similarly, we found that bortezomib, approved for the treatment of multiple myeloma and mantle cell lymphoma, was used off-label to treat acute lymphocytic leukemia. This use has been encouraged by studies that showed how the combination of bortezomib with chemotherapy is an effective option for the treatment of childhood relapsed/refractory acute lymphocytic leukemia (Bertaina et al., 2017; August et al., 2020). Finally, evidence on the off-label use of cyclophosphamide in the acute myeloid leukemia, melphalan in Wilms’ tumor, docetaxel in relapsed osteosarcoma, gemcitabine and vinorelbine in Hodgkin’s lymphoma have been found (Pein et al., 1998; Navid et al., 2008; Cole et al., 2009; Kobos et al., 2014; Song et al., 2014). Two ICSRs were categorized as off-label due to the use of brentuximab vedotin in a 16 years old patient affected by Hodgkin’s lymphoma. However, in the literature, it has been demonstrated that its pediatric use in relapsed or refractory Hodgkin’s lymphoma is well tolerated and associated with a positive clinical response (Locatelli et al., 2018). These findings are interesting because some drugs identified in the off-label ICSRs also have an orphan designation, which is evaluated by the Committee for Orphan Medicinal Products (COMP) of European Medicines Agency (EMA) and granted by the European commission for those medicines that are developed and authorized for a rare disease (European Medicine Agency, 2020). Is this the case for example of brentuximab vedotin that has the orphan designation for different onco-hematological diseases (European Medicine Agency, 2015; Orphanet, 2020). In Italy, orphan drugs are a topic of great importance and highly considered from regulators. In fact, since 2001, the Italian National Institute of Health (ISS, Istituto Superiore di Sanità) instituted the national registry on rare diseases (RNMR) with the aim of increasing the surveillance on rare disease, supporting national or regional planning in favor of patients with rare disease, improving the management of rare diseases, and favoring the debate among healthcare professionals for the definition of diagnostic criteria (Istituto Superiore di Sanità, 2001; Ministero della Sanità, 2001).

In our study, the majority of ADRs observed in off-label cases belong to the gastrointestinal and hematological systems. In the majority of these cases, it was also identified a drug-drug interaction among chemotherapy drugs that could explain the onset of gastrointestinal and hematological toxicities. One ICSR was related to the neurotoxicity induced by bortezomib. In the literature, this drug has been associated with the onset of peripheral neuropathy that often requires drug discontinuation (Meregalli, 2015). Moreover, in this ICSR was also identified an interaction between bortezomib and vincristine: both of them might increase the risk of nerve damage (Kassem et al., 2011). Finally, pharmacokinetics interactions between etoposide and fluconazole (N = 4), and sulfamethoxazole/trimethoprim and methotrexate (N = 1) were identified. It is known that fluconazole might increase the blood levels of etoposide, and sulfamethoxazole might increase the circulating levels of methotrexate. Hence, the increase of circulating active compounds might as well increase the frequency and severity of ADRs (Drugs.com).

We found that only one ICSR reported the term “off-label use” codified among ADRs. This is not surprising considering that a previous analysis conducted in our region has shown a low quality in ICSRs in terms of misclassified or unclassified cases for the non-normal use of drugs (like abuse or overdose) (Sessa et al., 2018a).

Finally, our study highlights the need for more researches on the off-label use of anticancer agents in children. We may say that there is little information on the efficacy/safety profile of these drugs and that often data are limited or confined to a specific age group, leading clinicians to the prescription of medicines at a dosage or for an indication that has not been approved in children.

### Strengths and Limitations

An important strength of our study is that ICSRs have been validated through a case-by-case approach by a team composed of clinicians and pharmacists well experienced in pharmacovigilance and clinical research. This guarantees good quality in a pharmacovigilance system and simplifies the recognition of off-label cases. Furthermore, we focused our analysis on ADRs occurred in children and adolescents receiving antineoplastic drugs. Considering that children with malignancies represent a frail population who is treated with very toxic medications and are at high risk for the occurrence of many ADRs, and given that the off-label use of medicines in the pediatric population is associated with high risk of developing ADRs too, we believe that the collection and analysis of data from the real life clinical practice, such as those deriving from the spontaneous reporting system, become essential for a better characterization of medicines especially in frail population, which was the main aim of our study. However, an important limitation is the lack of generalizability due to the small number of ICSRs of only one Italian region. Finally, our study has all the limitations of the spontaneous reporting system, such as underreporting, differential reporting, irregular information quality, and lack of denominator data such as the user population and drug-exposure patterns.

## Conclusion

This is the first study that aims to identify off-label ICSRs in oncology practice. However, we were able to identify only 18 ICSRs in which the anticancer agents were used off-label. The majority of ICSRs were categorized off-label for the “therapeutic indication.” Only two ICSRs were classified off-label due to the use of a drug for an unauthorized age. We believe that the low number of ICSRs classified as off-label is largely due to underreporting. However, spreading pharmacovigilance knowledge and awareness might improve this aspect. Therefore, we will start, in Campania region, several initiatives including pharmacovigilance active projects or specific pharmacovigilance courses for pediatric oncologists to stimulate and encourage the reporting of ADRs associated with the off-label use of anticancer drugs in children as pharmacovigilance data on this topic may fill in an important gap in knowledge. In order to improve a proper use of medicines in the pediatric population, collaborations between pediatricians, oncologists, and pharmacologists will be started.

## Data Availability Statement

The datasets generated for this study will not be made publicly available according to the Italian pharmacovigilance legislation. Datasets are only available for the Italian Medicine Agency or Pharmacovigilance regional centers.

## Ethics Statement

Ethical review and approval was not required for the study on human participants in accordance with the local legislation and institutional requirements. Written informed consent from the participants’ legal guardian/next of kin was not required to participate in this study in accordance with the national legislation and the institutional requirements.

## Author Contributions

Drafting the work and revising it for important intellectual content: AM, CS, MB, SB, FP, EP, MM, DP, FR. Substantial contributions to the acquisition, analysis, or interpretation of data for the work: AM, CS. Final approval of the version to be published: AM, CS, MB, SB, FP, EP, MM, DP, FR. Agreement to be accountable for all aspects of the work in ensuring that questions related to the accuracy or integrity of any part of the work are appropriately investigated and resolved: AM, CS, MB, SB, FP, EP, MM, DP, FR. Developed the concept and designed the study: FR. Wrote the paper: AM and CS.

## Conflict of Interest

The authors declare that the research was conducted in the absence of any commercial or financial relationships that could be construed as a potential conflict of interest.
