# Supplementary material for: Safety of Anticancer Agents Used in Children: A Focus on Their Off-Label Use Through Data From the Spontaneous Reporting System
Source: Front Pharmacol. 2020 May 7;11:621. doi: 10.3389/fphar.2020.00621 (PMC7221123; doi:10.3389/fphar.2020.00621)
Supplement: Supplementary file 1 [file Table_1.docx]

Supplementary Material

Article Title

**Safety of anticancer agents used in children: a focus on their off-label use through data from the spontaneous reporting system**

**Mascolo Annamaria^1*^ & Scavone Cristina^1^, Bertini Michele^1^, Brusco Simona^1^, Punzo Francesca^2^, Pota Elvira^2^, Di Martino Martina^2^, Di Pinto Daniela^2^, Rossi Francesca^2^**

1 Department of Experimental Medicine – Section of Pharmacology “L. Donatelli”, University of Campania “Luigi Vanvitelli”, Via Costantinopoli 16 – 80138 Naples (Italy)

2 Department of Woman, Child and General and Specialist Surgery University of Campania “Luigi Vanvitelli”, Via Costantinopoli 16 – 80138 Naples (Italy)

*** Correspondence:**Annamaria Mascolo
annamaria.mascolo@unicampania.it

**Keywords**: safety, pharmacovigilance, spontaneous reporting system, adverse drug reaction, anticancer agent, pediatric, off-label use.

**Supplementary table 1.** Most reported adverse drug reactions (ADRs) in non-off-label cases, distributed for the System Organ Class (SOC) in the setting of Campania spontaneous reporting system – January 2013 to September 2019.

| **SOC - Reported Adverse Events** | **Number** | **Percentage** |
| --- | --- | --- |
| **Preferred term** | **N** | **%** |
| **Gastrointestinal disorders** | **130** | **31.0** |
| *Vomit* | 29 | 22.3 |
| *Diarrhea* | 14 | 10.8 |
| *Nausea* | 13 | 10.0 |
| *Abdominal pain* | 14 | 10.8 |
| **Blood and lymphatic system disorders** | **64** | **15.2** |
| *Neutropenia* | 19 | 29.7 |
| *Anemia* | 13 | 20.3 |
| *Leukopenia* | 12 | 18.8 |
| *Thrombocytopenia* | 16 | 25.0 |
| **General disorders and administration site conditions** | **52** | **12.4** |
| *Pyrexia* | 18 | 34.6 |
| *Mucositis* | 12 | 23.1 |
| *Asthenia* | 8 | 15.4 |
| *Deterioration of general physical health* | 2 | 3.8 |
| **Respiratory, thoracic and mediastinal disorders** | **28** | **6.7** |
| *Oropharyngeal pain* | 8 | 28.6 |
| *Epistaxis* | 6 | 21.4 |
| *Productive cough* | 3 | 10.7 |
| *Pneumonia* | 2 | 7.1 |
| **Hepatobiliary disorders** | **26** | **6.2** |
| *Elevated transaminases* | 19 | 73.1 |
| *Hyperbilirubinemia* | 5 | 19.2 |
| *Hepatotoxicity* | 1 | 3.8 |
| *Jaundice of the sclera* | 1 | 3.8 |
| **Investigations** | **19** | **4.5** |
| *Antithrombin III decreased* | 4 | 21.1 |
| *Increased blood pressure* | 2 | 10.5 |
| *Positive copro-culture* | 2 | 10.5 |
| *Reduced ejection fraction* | 2 | 10.5 |
| **Skin and subcutaneous tissue disorders** | **18** | **4.3** |
| *Erythema* | 7 | 38.9 |
| *Petechiae* | 3 | 16.7 |
| *Rash* | 1 | 5.6 |
| *Hyperhidrosis* | 2 | 11.1 |
| **Metabolism and nutrition disorders** | **12** | **2.9** |
| *Hypertriglyceridemia* | 2 | 16.7 |
| *Hypoalbuminemia* | 2 | 16.7 |
| *Reduced appetite* | 2 | 16.7 |
| *Hyperlipasemia* | 2 | 16.7 |
| **Infections and infestations** | **14** | **3.3** |
| *Epididymitis* | 2 | 14.3 |
| *Device-related infection* | 2 | 14.3 |
| *Vulvovaginal candidiasis* | 1 | 7.1 |
| *Candidiasis* | 1 | 7.1 |
| **Nervous system disorders** | **14** | **3.3** |
| *Dizziness* | 5 | 35.7 |
| *Headache* | 3 | 21.4 |
| *Peripheral neuropathy* | 2 | 14.3 |
| *Pre-syncope* | 2 | 14.3 |
| **Musculoskeletal and connective tissue disorders** | **14** | **3.3** |
| *Limb pain* | 5 | 35.7 |
| *Spinal pain* | 3 | 21.4 |
| *Back pain* | 2 | 14.3 |
| *Bone pain* | 2 | 14.3 |
| **Vascular disorders** | **7** | **1.7** |
| Phlebitis | 2 | 28.6 |
| Hyperemia | 2 | 28.6 |
| Hematoma | 1 | 14.3 |
| *Hypertension* | 1 | 14.3 |
| **Congenital, familial and genetic disorders** | **5** | **1.2** |
| *Aplasia* | 5 | 100.0 |
| **Cardiac disorders** | **4** | **1.0** |
| *Sinus tachycardia* | 2 | 50.0 |
| *Heart failure* | 1 | 25.0 |
| *Palpitation* | 1 | 25.0 |
| **Renal and urinary disorders** | **4** | **1.0** |
| *Acute renal failure* | 2 | 50.0 |
| *Hemorrhagic cystitis* | 1 | 25.0 |
| *Hematuria* | 1 | 25.0 |
| **Reproductive system and breast disorders** | **3** | **0.7** |
| *Vulvovaginal itching* | 2 | 66.7 |
| *Testicle pain* | 1 | 33.3 |
| **Eye disorders** | **2** | **0.5** |
| *Conjunctival hyperemia* | 1 | 50.0 |
| *Increased lachrymation* | 1 | 50.0 |
| **Psychiatric disorders** | **2** | **0.48** |
| *Agitation* | 1 | 50.00 |
| *Mood alteration* | 1 | 50.00 |
| **Immune system disorders** | **1** | **0.2** |
| *Hypogammaglobulinaemia* | 1 | 100.0 |
| *Injury, poisoning and procedural complications* | 1 | 0.2 |
| *Off-label use* | 1 | 100.0 |
| **Total** | **420** | **100.0** |
